# The Influence of the Ketogenic Diet on the Immune Tolerant Microenvironment in Glioblastoma

**DOI:** 10.3390/cancers14225550

**Published:** 2022-11-11

**Authors:** Pravin Kesarwani, Shiva Kant, Yi Zhao, C. Ryan Miller, Prakash Chinnaiyan

**Affiliations:** 1 Department of Radiation Oncology, Beaumont Health, 3811 West Thirteen Mile Road, Royal Oak, MI 48073, USA; 2 Department of Pathology at UAB, Birmingham, AL 35233, USA; 3 O’Neal Comprehensive Cancer Center and Comprehensive Neuroscience Center, Birmingham, AL 35233, USA; 4 Radiation Oncology, Oakland University William Beaumont School of Medicine, Royal Oak, MI 48309, USA

**Keywords:** ketogenic diet, GBM, immunosuppressive macrophages, CSF-1R inhibition

## Abstract

**Simple Summary:**

Preclinical investigations have identified promising anti-tumor activity of a ketogenic diet in glioblastoma. Although a majority of the work has focused on how this diet directly influences tumor growth, our understanding of its impact on the tumor microenvironment remains limited. In this study, although the anti-tumor activity of a ketogenic diet in glioblastoma was validated, we uncovered a paradoxical increase in a “pro-tumoral” immune response, skewing tumor-associated macrophages towards an “M2-like” phenotype. We hypothesized this would limit the anti-tumor activity of a ketogenic diet, which was tested by combining this diet with a CSF-1 inhibitor. The combination of a ketogenic diet with CSF-1 inhibition normalized tumor-associated macrophages and led to a profound improvement in survival in mice. This provides rationale to test this novel combinatorial strategy in glioblastoma and highlights the importance of understanding how a given therapy influences both the tumor and its associated immune microenvironment.

**Abstract:**

Glioblastoma (GBM) represents an aggressive and immune-resistant cancer. Preclinical investigations have identified anti-tumor activity of a ketogenic diet (KD) potentially being used to target GBM’s glycolytic phenotype. Since immune cells in the microenvironment have a similar reliance upon nutrients to perform their individual functions, we sought to determine if KD influenced the immune landscape of GBM. Consistent with previous publications, KD improved survival in GBM in an immune-competent murine model. Immunophenotyping of tumors identified KD-influenced macrophage polarization, with a paradoxical 50% increase in immune-suppressive M2-like-macrophages and a decrease in pro-inflammatory M1-like-macrophages. We recapitulated KD in vitro using a modified cell culture based on metabolomic profiling of serum in KD-fed mice, mechanistically linking the observed changes in macrophage polarization to PPARγ-activation. We hypothesized that parallel increases in M2-macrophage polarization tempered the therapeutic benefit of KD in GBM. To test this, we performed investigations combining KD with the CSF-1R inhibitor (BLZ945), which influences macrophage polarization. The combination demonstrated a striking improvement in survival and correlative studies confirmed BLZ945 normalized KD-induced changes in macrophage polarization. Overall, KD demonstrates antitumor activity in GBM; however, its efficacy is attenuated by promoting an immunosuppressive phenotype in macrophages. Combinatorial strategies designed to modulate macrophage polarization represent a rational approach to improve the anti-tumor activity of KD in GBM.

## 1. Introduction

Glioblastoma (GBM) is the most common primary malignant brain tumor in adults, accounting for more than half of all diagnosed brain cancers [1]. Despite advancements in both surgical and radiation techniques, clinical outcome remains poor and long-term survival rare [1]. Although there has been an abundance of novel targeted therapies designed to treat cancer in recent years, a majority of these agents have a limited ability to cross the blood–brain barrier (BBB) and achieve clinically relevant concentrations in the brain, limiting their application in GBM [2]. Further, although immunotherapies have changed the therapeutic landscape of cancer, clear anti-tumor activity has yet to be observed in GBM [3]. Therefore, novel therapeutic approaches and/or application of currently available agents are needed to further clinical gains in this malignancy.

Diet modification and nutritional supplementation has gained considerable attention as an approach to both preventing and treating a variety of illnesses, including cancer [4,5]. The ketogenic diet (KD), which consists of a high-fat, low-carbohydrate diet, represents one of the most studied approaches, being described as a strategy to treat epileptic seizures in the early 1900s [6]. Numerous preclinical investigations have demonstrated promising anti-tumor activity of a KD in a diverse panel of cancers, including GBM [5]. Based on the limitations the BBB places on systemic agents’ ability to enter the central nervous system (CNS), the potential for diet modification to have anti-tumor activity makes the KD particularly appealing in brain tumors.

A commonly ascribed mechanism underlying the anti-tumor activity of a KD relates to its ability to target the Warburg effect, which is characterized by a bioenergetic shift observed in cancer cells from oxidative phosphorylation and reliance upon glycolysis to fuel the energetic needs of these cells [7,8]. Therefore, it has been postulated that, based on the very low levels of carbohydrates present in this diet, the KD has the capacity to “starve” cancer cells of their requisite glucose [7,9]. Although a seemingly logical approach, recent investigations suggest additional layers of complexity and regulation between the KD and general glucose metabolism. This includes studies demonstrating unchanged concentrations of blood glucose despite being on this diet [10,11], which would challenge low glucose levels as the primary mechanism underlying its anti-tumor activity. A variety of alternate hypotheses underlying the anti-tumor activity of the KD have been proposed, including ketone bodies, reactive oxygen species (ROS), and epigenetic modulation [12,13].

Although mechanisms underlying the anti-tumor activity of a KD remain an active area of investigation, the anti-inflammatory properties of this diet appear to be a consistent theme along multiple lines of investigation [14,15]. Inflammation represents a central component of oncogenesis [16,17] and is largely mediated by immune cells in the tumor microenvironment. Accordingly, there have been several publications describing the capacity of a KD to modulate the presence and function of a variety of immune components [18,19,20,21]. Although immune evasion represents a hallmark of cancer [17], and there appears to be a clear link between a KD and immune response, studies directly evaluating the influence of this diet on the immune microenvironment of cancer are limited. In this study, we sought to determine how a KD may influence immune tolerance in GBM, which represents a particularly immune-suppressive tumor. Through these studies, we uncovered a previously undescribed ability of this diet to modulate macrophage polarization. Although the anti-tumor activity of a KD was observed in a mouse GBM model, this activity appeared to be tempered by a paradoxical skewing of macrophage polarization towards a “pro-tumorigenic” phenotype. Combinatorial strategies designed to inhibit macrophage polarization demonstrated a significant improvement in survival in mice fed a KD, providing a framework to extend clinically.

## 2. Materials and Methods

### 2.1. Animal Studies

Animal studies were conducted in an AAALAC-accredited facility under animal protocols AL-18-10, AL-2021-04, and AL-19-07. All animal experiments were approved by the IACUC at Beaumont Research Institute. C57BL/6 and Nu/Nu mice were purchased from Charles River Laboratories (Wilmington, MA, USA). Animal experiments were conducted with an equal number of age- and sex-matched (6–12 weeks old male and female) mice.

### 2.2. Cell Culture and Reagents

The genetically engineered murine tumor model (TRP) GBM line was generated from genetically engineered mice (GEM) and cultured in Minimum Essential Media (MEM) as previously described [22,23,24]. Mesenchymal (MES83) GBM tumor-initiating cells were generated, obtained, authenticated, and provided by Dr. Nakano [25]. Primary cell cultures of murine T cells and macrophages were performed in complete RPMI-1640. Description of the reagents and antibodies used in the study is detailed in the Supplementary Methods.

### 2.3. Ketogenic Diet

The ketogenic diet (TD.130659; Envigo Teklad Diets, Madison, WI, USA) comprises 15.1% protein, 2.4% carbohydrate, and 72.2% fat by weight, yielding a fat to protein+carbohydrate ratio of approximately 4:1. Fats in this KD yield about 90.3% (Kcal) of energy. Protein and carbohydrates yield about 1.4% and 8.4%, respectively. Standard rodent chow yields 24% calories from protein, 60% from carbohydrate, and only 16% from fats.

### 2.4. Macrophage Polarization

Bone marrow cells were isolated from the femurs of C57BL/6 mice and cultured in CSF (40 ng/mL) for 5–6 days to generate M0 cells. Cells were then cultured with IL-4 (20 ng/mL) and IL-13 (20 ng/mL) for M2 polarization or IFN-γ (50 ng/mL) and LPS (100 ng/mL) for M1 polarization (2 d). On day 3, 200 μM of linoleic acid (LA) or oleic acid (OA) were added and maintained at the same level throughout polarization and activation. PPARγ inhibitor (GW9662) was added during polarization at the concentration of 0.5 μM and maintained throughout polarization and activation.

### 2.5. Global Metabolomic Analysis

Nu/Nu mice were injected with human MES83 tumors. In addition, a group of mice received a ketogenic diet. Blood was extracted from these mice on day 13 from the start of the ketogenic diet. Serum was extracted from these samples and flash frozen. Targeted global metabolomic profiling was performed for more than 900 metabolites associated with carbohydrates, lipids, amino acids, peptides, nucleotides, cofactors, and vitamins, with analysis by Metabolon Inc. using liquid or gas chromatography-mass spectrometry (LC-MS or GC-MS) methods described previously [26].

### 2.6. Flow Cytometry

A complete description of flow cytometry is given in the Supplementary Methods section. Briefly, cells from tumor or in vitro cultures were stained for cell surface proteins and nuclear proteins. An equal number of cells from each sample were acquired on a FACS Canto II flow cytometer (BD; Mountain View, CA, USA). Analysis of flow cytometry data was performed using FlowJo V10 software (FlowJo, LLC; Ashland, OR, USA). *Gating strategy:* We used expression of CD11b and/or CD45 for the initial gating of three immune subsets: (i) macrophages (CD45+CD11b+), (ii) lymphocytes (CD45+CD11b-ve), and (iii) microglia (CD45^Medium^CD11b+). Using the initial macrophage gate, we further analyzed M2 macrophages (CD45+CD11b+ F4/80+CD206++CD80^Low^), Arginase 1+ M2 macrophages (CD45+CD11b+ F4/80+CD206+Arginase 1+), M1 macrophages (CD45+CD11b+F4/80+CD206-ve, CD80^High^), iNOS+M1s (CD45+F4/80+CD11b+CD206-ve, CD80hi+iNOS+), and MDSCs (CD45+ CD11b+ Gr1+). In lymphocyte gates we analyzed CD4 T lymphocytes (CD45+CD4+), CD8 T lymphocytes (CD45+CD8+), Tregs (CD45+CD4+CD25+FoxP3+), CD8+GzmB+ T lymphocytes (CD45+CD8+GzmB+), and NK cells (CD45+NK1.1+NKG2D+). Microglia were defined as CD45^medium^, CD11b+CD68+.

### 2.7. Statistical Analysis

Comparisons across two groups were performed on original data using a two-tailed Student’s *t*-test. Comparison between more than two groups was conducted using ANOVA followed by post hoc comparisons. A Wilcoxon signed-rank test was used to analyze the difference between the global metabolic profile of serum between the two groups. A log-rank test was used for survival analyses. The differential abundance score (DA) was calculated using metabolites significantly (Mann-Whitney U tests and Benjamini-Hochberg corrected *p* value < 0.05) increased or decreased in the KD group in comparison to the SD group. Metabolites were classified into major metabolic pathways. Metabolic pathways with a minimum of 4 significantly modulated metabolites were chosen for this plot. Volcano plots were used to show the difference between metabolite fold changes between KD and SD. For generating volcano plots, we used metabolite values to calculate the *p*-value. Fold changes were converted to negative log2 (*x*-axis) and compared to negative log10 of *p* value (*y*-axis). The metabolites in the volcano plot were classified into carbohydrates, lipids, amino acids, nucleotides & peptides, cofactors, and vitamins. Metabolites with a *p* value less than 0.05 and fold change above 2 or below 0.5 were represented with a solid circle. A pie chart was calculated using the number of significantly upregulated lipids based on their saturation levels (double bond). Lipids with double bonds in the backbone or hydrocarbon chains were considered unsaturated for the pie chart analysis. All statistical analyses were performed using Origin Pro 2020 software (Origin Lab Corporation; Northampton, MA, USA).

### 2.8. Supplementary Methods 

The Supplementary Methods section contains a detailed methodology for following experimental assays, establishment of orthotopic tumor models, PPARγ transcriptional activation assay, CD8 T cell suppression assay, flow cytometry, blood glucose and ketone analysis, and Western blotting.

## 3. Results

**The ketogenic diet skews macrophage polarization towards a “pro-tumorigenic” M2-like phenotype.**

**Figure 1:**
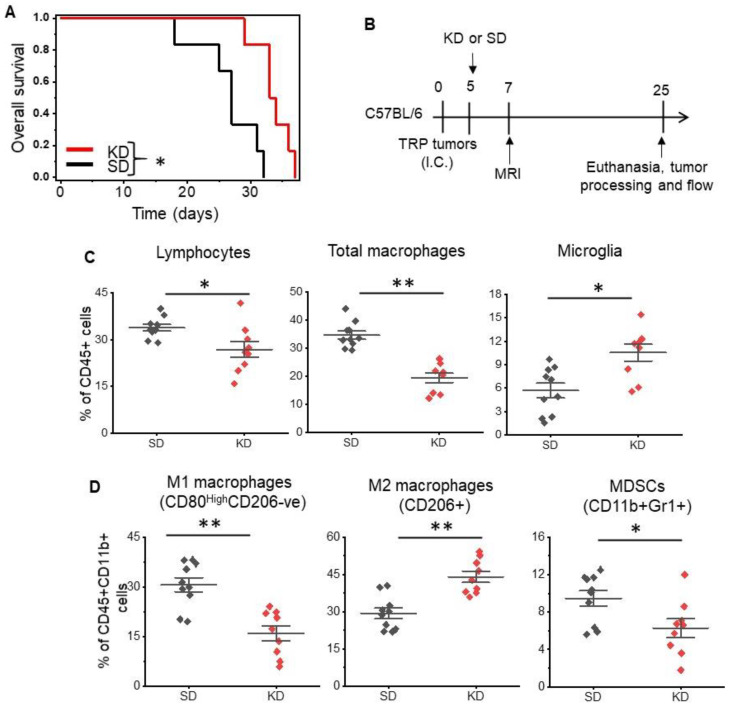
**The ketogenic diet modulates macrophage polarization in GBM.** Mouse-derived genetically engineered GBM cells (TRP) were grown orthotopically in immune-competent C57BL/6 mice fed a standard rodent diet (SD) rodent diet or a ketogenic diet (KD) ad libitum. (**A**) Survival analysis between KD and SD. n = 6/group. (**B**) The schema used for correlative studies. (**C**) Tumors harvested from KD and SD fed mice were immunophenotyped, evaluating for total lymphocytes (CD45+CD11b-ve), total macrophages (CD45+CD11b+), and microglial cells (CD45^medium^CD11b+), (**D**) M1 macrophages (CD45+F4/80+CD11b+CD80^High^CD206-ve), M2 macrophages (CD45+CD11b+F4/80+CD206+CD80^Low^), and myeloid-derived suppresser cells (MDSCs) (CD45+CD11b+Gr1). The line between the data points represents the mean, and the whisker represents SE. n = 9–10/group. *: *p* < 0.05, **: *p* < 0.005.

We evaluated the anti-tumor activity of a KD in vivo using a novel, adult astrocytic, genetically engineered mouse-derived (GEM) cell line that has been shown to recapitulate both the molecular and histopathological features of human GBM [22,23,24]. The systemic effects of a KD were validated in serum, as demonstrated by elevated levels of ketones in mice fed a KD (Figure S1A), although glucose levels remained unchanged between the two groups (Figure S1B). Consistent with previous reports [10,27] changing diet alone led to anti-tumor activity in mice, with a KD demonstrating improved median and overall survival in this immune-competent GBM orthotopic model (Figure 1A).

Next, using flow cytometry, we immunophenotyped tumors harvested from mice fed a standard diet (SD) and KD as an initial investigation to determine if a KD influenced the immune microenvironment in GBM (Figure 1B). Initial studies focused on total macrophages (CD45+F4/80+CD11b+), microglia (CD11b+CD45^Medium^CD68+), and lymphocytes (CD45+CD11b-ve), and demonstrated significant changes in the immune profiles of these tumors. Specifically, decreases in both total lymphocytes (33.8 ± 7.6% vs. 26.9 ± 3.3%; *p* value < 0.01) and macrophages (34.7 ± 5% vs. 19.4 ± 4.6%; *p* value < 0.0005) and an increase in microglia (5.7 ± 3% vs. 10.7 ± 3.2%; *p* value < 0.003) were observed in mice fed a KD (Figure 1C).

As recent data suggest, macrophages play a central role in the immune-tolerant microenvironment in GBM [28,29,30], and as the KD appeared to influence their levels in our GBM model, we evaluated the interaction between diet and macrophage polarization in further detail. Using CD45+F4/80+CD11b+ gating, we further analyzed several subsets of macrophages, including M1-like macrophages (CD45+CD11b+F4/80+CD80^high^CD206-ve), M2-like macrophages (CD45+CD11b+F4/80+CD206+CD80^low^), and myeloid-derived suppresser cells (MDSCs). Interestingly, when evaluating for specific macrophage subtypes, we observed a considerable shift in macrophage polarization following a KD (Figure 1D). There was a ~50% reduction in M1-like macrophages (30.6 ± 6.8% vs. 16 ± 6.9%; *p* value < 0.002) and ~50% increase in M2-like macrophages (29.4 ± 6.3% vs. 44 ± 6.8%; *p* value < 0.003), suggesting that although demonstrating anti-tumor activity, in parallel, the KD may also support tumor growth by further promoting the immune-suppressive microenvironment. Although MDSCs represent a smaller fraction of the three myeloid subsets, there also appeared to be an increase in MDSCs in the KD group (9.5 ± 2.6% vs. 6.3 ± 3.0%; *p* value < 0.026). When evaluating T cell components, there were no changes in CD8+ T cells or T regulatory cells (Tregs); however, modest decreases in CD4+ T cells were observed in mice fed a KD (Figure S2; 48.6 ± 5.9% vs. 39 ± 5.4%; *p* value < 0.002). In addition, an unexpected increase in natural killer (NK) cells was observed in KD mice (Figure S2; 14.5 ± 2.2% vs. 25.8 ± 5.3%; *p* value < 0.0001).

**Fatty acids promote M2 macrophage polarization through PPARγ signaling.**

**Figure 2:**
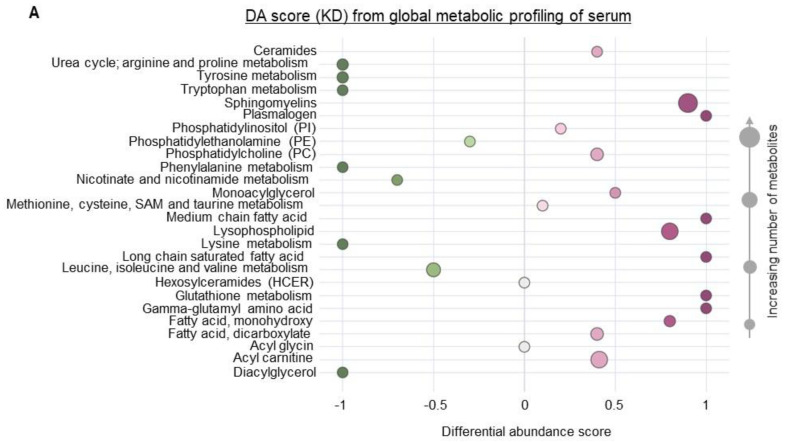

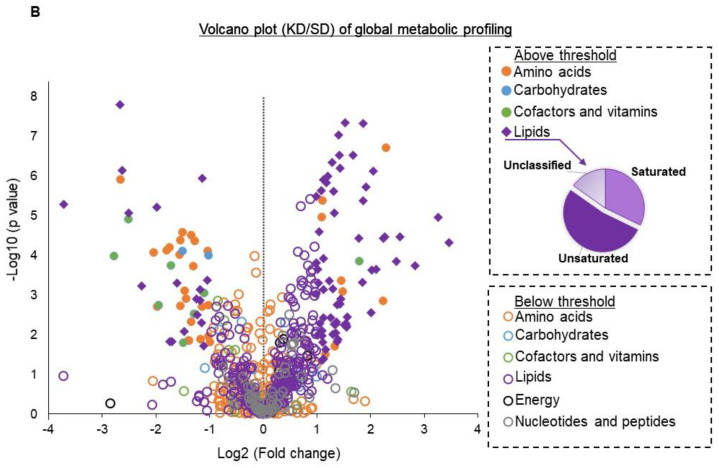
**Systemic metabolic changes associated with a ketogenic diet.** Global metabolic profiling was performed on serum from mice fed a KD or SD (n = 6/group). (**A**) Significantly altered metabolites were classified into major metabolic pathways, and the differential abundance (DA) scores of each pathway were plotted. (**B**) Metabolic profiles of KD and SD were classified into lipid, nucleotides and peptides, amino acids, carbohydrates, and cofactor and vitamin metabolism. Values from each metabolite were used to calculate log2 (fold change KD/SD) and plotted against—Log10 (*p*-value) to generate a volcano plot. Metabolites with >2-fold change and with a *p* value < 0.05 were considered to be above the threshold (solid circles or diamonds). Metabolites with <2-fold change or with a *p* value > 0.05 were below the threshold (empty circles). Lipids with significant *p* values were characterized into 3 groups (saturated, unsaturated, and unclassified) based on saturation levels and their abundance and depicted as a pie chart.

To begin to understand how a KD influences the immune profile of GBM, we performed metabolomic profiling on serum obtained from mice fed a SD or KD to provide a global perspective on the systemic changes associated with diet modification. These studies uncovered numerous metabolic pathways differentially regulated in mice fed a KD. Interestingly, amino acid metabolism, including metabolites associated with the urea cycle, tryptophan, and phenylalanine, represented one of the most strongly attenuated metabolic pathways in KD fed mice (Figure 2A,B). Although a KD consists of a high fat and low carbohydrate diet, we expected these dietary lipids to be rapidly metabolized and/or stored, resulting in the normalization of plasma concentrations of these metabolites. However, our findings identified high levels of lipids remaining in the plasma, representing one of the most dominant metabolic pathways differentially regulated in a KD, with most of these lipids being unsaturated fatty acids (Figure 2A,B).

**Figure 3:**
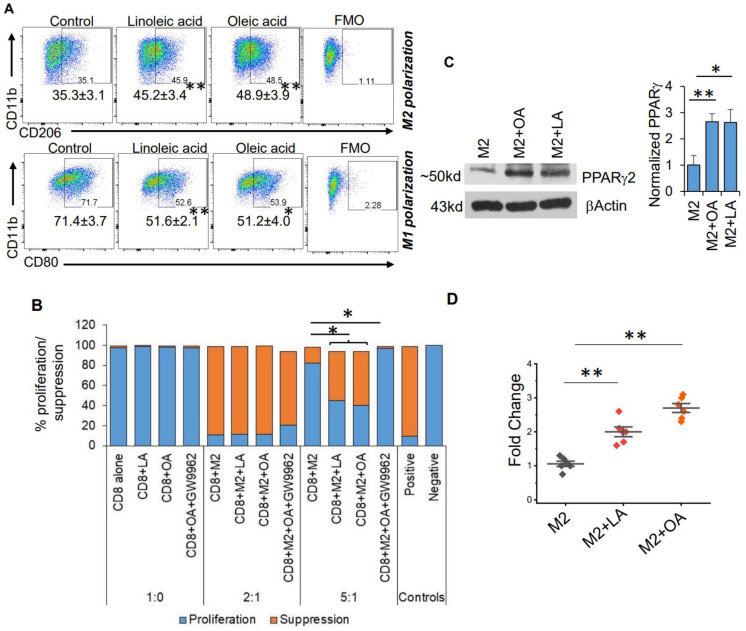

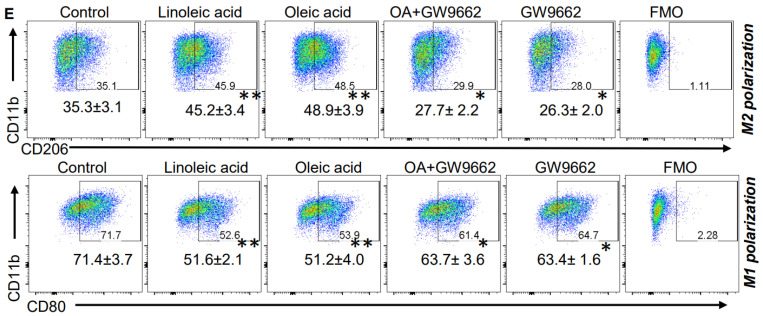
**Fatty acids modulate macrophage polarization.** (**A**) Myeloid cells were obtained from the bone marrow of C57BL/6 mice and polarized towards M2 macrophages or M1 macrophages ± the indicated unsaturated fatty acid (linoleic acid [LA] 200 mM; oleic acid [OA] 200 µM) and analyzed via flow cytometry. Numbers represent mean±SD from a minimum of 3 different experiments. (**B**) To analyze the functional aspect (immune suppressive ability) of M2 macrophages, mouse-derived M2 cells were generated as described in (**A**) and polarized with indicated agents. Splenocytes from C57BL/6 mice were used for isolating CD8+ T cells with magnetic bead sorting. CFSE labeled CD8+ T cells were activated using plate-bound anti-CD3/CD28 antibodies for three days in the presence or absence of M2 cells. Data are represented as a bar graph demonstrating CFSE dilution (proliferation) or suppression. CFSE labeled CD8+ T cells without stimulation (anti-CD3/CD28 antibody) were used as a positive control of suppression. Unlabeled CD8+ T cells were used as a negative control for proliferation. (**C**) Mouse bone-marrow-derived M2 macrophages were cultured in ± LA (200 mM) or OA (200 µM) and analyzed for indicated proteins using Western blot. Bar graph (n = 3 experiments) represents PPARγ protein normalized to βActin. (**D**) PPARγ transcriptional activity was evaluated in M2 macrophages cultured ± LA (200 mM) or ±OA (200 µM). (**E**) Myeloid cells were obtained from the bone marrow of C57BL/6 mice and polarized towards M2 macrophages or M1 macrophages ± the indicated unsaturated fatty acid (linoleic acid [LA] 200 mM; oleic acid [OA] 200 µM) ± PPARγ antagonist GW9662 and analyzed using flow cytometry. Data are representative of a minimum of three experiments. *: *p* < 0.05, **: *p* < 0.005.

Based on these metabolomic studies, which demonstrate a robust accumulation of fatty acids in the serum of mice fed a KD, we sought to determine if these systemic metabolic effects could influence the observed changes in macrophage plasticity. As an initial investigation, to try to recapitulate in vivo findings in vitro, we polarized mouse-derived monocytes towards the M1 and M2 macrophage phenotype in the presence or absence of the unsaturated fatty acids linoleic and oleic acid. Consistent with what was observed in mice fed a KD, culturing monocytes in the presence of these fatty acids modulated macrophage polarization, with significant increases in M2-like macrophage polarization and attenuated M1-like macrophage polarization (Figure 3A). We also found that M2 macrophages cultured in the presence of unsaturated fatty acids linoleic and oleic acid had higher Arginase 1 (Figure S3), suggesting a higher immunosuppressive ability of M2 macrophages. We went on to determine if the observed fatty acid-induced changes in M2 macrophage polarization also influenced the capacity of these cells to functionally suppress CD8+ T-cell proliferation using CD8/M2 macrophage co-culture experiments. At the indicated CD8:M2 ratio (5:1), M2 macrophages demonstrated modest inhibition of CD8 cell proliferation (~20%). Consistent with polarization studies, M2 macrophages cultured in the presence of both oleic and linoleic acid led to potent inhibition of CD8+ T cell proliferation (~60%; Figure 3B). These findings collectively support global metabolic alterations associated with a KD, resulting in an accumulation of fatty acids and potentiating the immune tolerant microenvironment in GBM by skewing macrophage polarization towards a “pro-tumorigenic” phenotype.

We went on to determine mechanisms contributing toward fatty-acid-induced modulation of macrophage polarization. We tested the hypothesis that the transcription factor PPARγ, previously described as regulated by fatty acids [31] and an established mediator of macrophage polarization [32,33], contributed toward the observed immunologic changes associated with a KD. A Western blot performed using bone-marrow-derived M2 macrophages demonstrated increased expression of PPARγ when cultured in the presence of both oleic and linoleic acid (Figure 3C), supporting a link between a KD and activation of this transcriptional program. This was further validated using a PPARγ -specific transcriptional activation assay (Figure 3D). To further support the hypothesis that fatty acids mediate macrophage polarization through PPARγ signaling, we utilized the PPARγ inhibitor GW9662. In addition to normalizing linoleic and oleic acids’ influence on both M2 and M1 polarization (Figure 3E), GW9662 inhibited the functional suppression of these M2 macrophages (Figure 3B), further supporting the contributory role of this signaling axis in the observed changes of macrophage polarization.

**Mitigating KD-induced changes in macrophage polarization through CSF-1 inhibition potentiates anti-tumor activity.**

**Figure 4:**
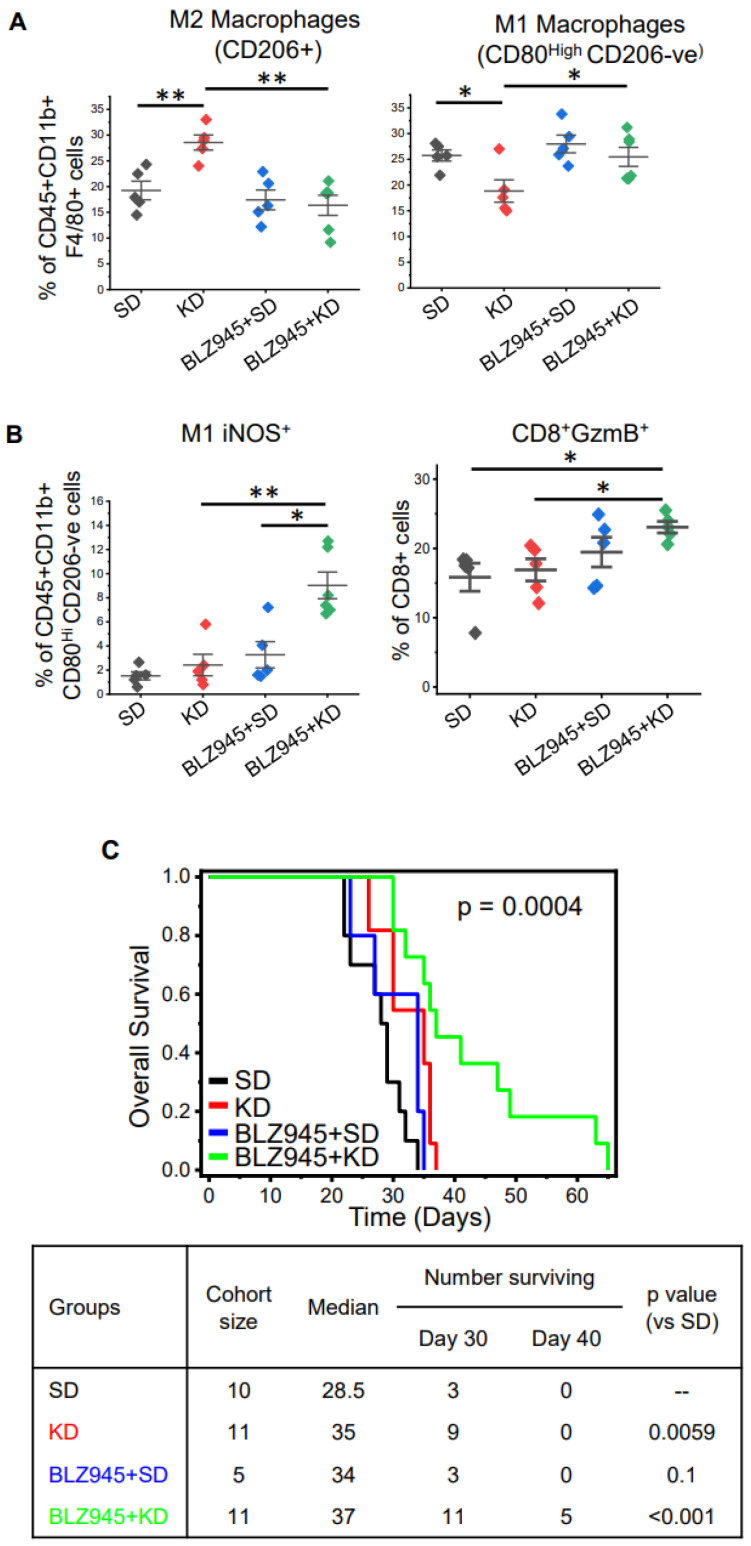
**Targeting macrophage polarization through CSF-1 inhibition enhances the anti-tumor activity of a KD.** TRP tumors were grown orthotopically in immune-competent C57BL/6 mice. Tumor establishment was confirmed using MRI. Mice were fed a standard diet (SD) or ketogenic diet (KD) ± CSF-1 inhibitor BLZ945. (**A**,**B**) Tumors were harvested on day 25 and immunophenotyped for indicated cells via flow cytometry (n = 5–6/group). (**C**) Kaplan–Meier survival plot in mice treated with the indicated agents (n = 7–10/group). Table describes cohort size, median survival, number of mice surviving on days 9, 30, and 40, and log rank *p* value between different groups when compared to SD group. The line between the data points represents the mean, and the whisker represents SE. *: *p* < 0.05, **: *p* < 0.005.

Although we demonstrated clear anti-tumor activity following a KD in GBM, we hypothesized that parallel increases in immunosuppression through M2 macrophage polarization mitigated the therapeutic potential of this diet. Macrophages require colony-stimulating factor 1 (CSF-1) to attain an M2-like immunosuppressive phenotype, and CSF-1 receptor inhibition represents a promising strategy to attenuate M2-like macrophages in GBM [34,35]. We therefore sought to determine if CSF-1 inhibition could enhance the anti-tumor activity of a KD by normalizing this paradoxical increase in immune suppression. To test this, we performed in vivo studies evaluating mice fed a SD or KD in combination with and without the CSF-1R inhibitor BLZ945. Similar to our initial studies described above, immunophenotyping of tumors extracted from mice fed a KD demonstrated lower levels of lymphocytes and macrophages and an increase in microglial cells (Figure S4A,B). The addition of BLZ945 alone or in combination with a KD did not appear to influence these profiles. Although CSF-1R inhibition did not regulate levels of total macrophages in the tumor microenvironment, intriguingly, it did demonstrate the capacity to normalize KD-induced changes in macrophage polarization. Specifically, previously described KD-induced increases in M2-like macrophages and decreases in M1-like macrophages were normalized when combined with BLZ945 (Figure 4A). Although no changes in CD4 or CD8 cells were observed (Figure S4C), a significant increase in M1-like macrophages with iNOS (inducible nitric oxide synthase) was demonstrated with the BLZ945/KD combination (Figure 4B), suggesting that inhibition of M2 signaling switches the profile of suppressive macrophages to a proinflammatory phenotype when combined with a KD. In addition, an increase in CD8+GzmB+ T cells was observed with this combination, suggesting an increase in immune activation. When evaluating for survival, consistent with our hypothesis, the combination of CSF-1R inhibition and KD demonstrated an improvement in overall survival, including ~50% of mice treated with this combination staying alive when all mice in the remaining three treatments required sacrifice secondary to progressive disease (Figure 4C).

## 4. Discussion

Although nearly a century has passed since the KD was utilized as a treatment for childhood epilepsy, its roots date back thousands of years, being developed as a strategy to mimic a metabolic state of fasting that may be maintained for extended periods of time [36]. Relative to this long history, the majority of work evaluating the application of the KD as a form of cancer therapy only spans a decade. The initial rationale for extending this diet to treat cancer is its potential to target the glycolytic phenotype of cancer [7,8]. Accordingly, the primary focus of these studies involved how this diet could directly influence cancer cell growth. Research evaluating how a KD influences the tumor microenvironment (TME), which is also highly dependent on the availability of macro- and micronutrients, is limited. This is particularly relevant in cancer, as recent work has underscored the critical role the TME plays in immune evasion and therapeutic resistance [17]. Based on previous reports demonstrating the potential of a KD to attenuate inflammation [21,37], we sought to determine if this diet modulated the immune microenvironment in GBM, which represents a particularly immune-suppressive tumor. Paradoxical to the observed anti-tumor activity of a KD in GBM, this diet potentiated immune suppression by skewing tumor-associated macrophages towards a “pro-tumorigenic” phenotype. Specifically, mice fed a KD demonstrated a 50% increase in immune suppressive M2-like macrophages and a 50% reduction in M1-like macrophages, which have been demonstrated to stimulate inflammation and target cancer cells. In addition to quantitively increasing M2 macrophages in the TME, KD influenced the function of these cells, making them more immune suppressive. These findings are particularly relevant in GBM, as M2-like macrophages appear to play a central role in the potent immune suppression observed in this malignancy [38,39,40].

Although the KD derived its name for its ability to induce an accumulation of ketone bodies in the blood, including beta-hydroxybutyrate, which may directly or indirectly play a role in its therapeutic efficacy, its application in cancer is largely based on the constituents of the diet itself. Specifically, the very low proportion of carbohydrates in the KD could theoretically “starve” highly glycolytic cancer cells [7,8]. Consistent with previous studies, although ketosis was validated in our preclinical model, blood glucose concentrations in mice fed a KD remained unchanged when compared to mice fed a SD. Based on these findings, the anti-tumor activity conferred by this diet is likely independent of carbohydrate restriction in our model. To gain a broader understanding of the systemic consequences of a KD and to identify factors that may be contributing towards KD-induced macrophage polarization, we performed high-throughput metabolomic profiling, comparing mice fed a SD with a KD. Through these studies, although the differential activity of several intriguing metabolic pathways was uncovered in mice fed a KD, the most dominant was a high concentration of serum fatty acids. Based on the unchanged concentrations of blood glucose in KD mice, we expected to observe a similar normalization of diet-rich fatty acids through rapid utilization and/or lipid storage mechanisms. The high levels of fatty acids in the blood, primarily consisting of unsaturated fatty acids, prompted us to determine if these could play a more direct role in the observed effects of KD on the TME. Intriguingly, culturing mouse-derived macrophages in unsaturated fatty acids stimulated M2 macrophage polarization and attenuated M1 macrophage polarization in a manner consistent with what was observed in vivo in mice fed a KD. Additionally, we found that fatty acids increased Arginase 1+ macrophages in vitro. It is well known that Arginase 1 increases the immunosuppressive function of tumor-associated macrophages (TAM) [41]. A recent study established that GBM tumors produce exosomes in the tumor microenvironment converting M1-type macrophages to “M2-like” TAMs. These “M2-like” TAMs produce Arginase-1+ exosomes in the tumor microenvironment, inducing the proliferation of GBM tumors, further augmenting pro-tumor functions [42]. In addition to skewing macrophage polarization towards an M2-like phenotype, fatty acids conferred potent immune suppressive properties to these macrophages, which was determined through co-culture experiments demonstrating diminished T cell proliferation. We went on to mechanistically determine that fatty-acid-induced changes in macrophage polarization were mediated through PPARγ signaling, which represents a pathway previously described to both be modulated by fatty acids and contribute towards M2 macrophage polarization [32,33]. Studies have also demonstrated that high PPARγ activity is also associated with a consequent increase in arginase 1 expression, suggesting KD-associated fatty acids may have similar effects on the immunosuppressive function of tumor resident macrophages [43].

Based on these seemingly paradoxical findings of anti-tumor activity in a KD despite contributing towards a “pro-tumor” immune response, we hypothesized the observed immune response tempered the therapeutic benefit of a KD, and normalizing this would further improve the anti-tumor activity of this approach. To test this, we performed combinatorial studies utilizing the colony-stimulating factor-1 receptor (CSF-1R) inhibitor BLZ-945. CSF-1 plays an important role in macrophage development and differentiation. The binding of CSF-1 to CSF-R1 triggers auto-phosphorylation of the receptor and activates multiple intracellular pathways, resulting in macrophage maturation and the upregulating of genes contributing toward the M2-like macrophage phenotype [44]. As M2-like macrophages are considered to be one of the key factors contributing toward immune tolerance in GBM [28,40,45], targeting the CSF-R1 receptor axis has been an active area of research. Unfortunately, as our preclinical studies suggested, targeting this pathway alone did not result in anti-tumor activity, although combination studies using this agent appear promising. This would suggest that approaches designed to independently target the established immune microenvironment in GBM, as it relates to M2 macrophages, may be limited, although combinations that include strategies designed to disrupt this environment, forcing macrophage re-polarization (e.g., radiation therapy) may hold promise. Consistent with our previous work, BLZ-945 alone did not influence the immune landscape in our mouse model of GBM or result in anti-tumor activity [34]. However, as hypothesized, in combination, BLZ-945 normalized KD-induced changes in macrophage polarization and triggered immune activation, resulting in a significant improvement in survival.

The immune system is composed of two parts, innate and adaptive immune components. Innate immune cells act swiftly, as they have an innate ability to recognize a limited repertoire of germline-encoded receptors. In contrast, adaptive immune cells (T cells and B cells) can identify an extensive repertoire of antigen receptors produced by site-specific somatic recombination. It is well known that a combination of innate and adaptive immune responses is critical to elicit an efficient anti-tumor response. Our study establishes that a KD influences several innate immune cells, such as macrophages, MDSCs, and NK cells. Although a KD demonstrates anti-tumor activity, it appears tempered by an increase in immunosuppressive M2-like macrophages and a reduction in pro-inflammatory M1-like macrophages. CSF-1R inhibition both restored this balance and increased iNOS+ M1-like macrophages, which produce toxic nitric oxide that lyses tumor cells and promotes phagocytosis [46]. Our study also demonstrated a marked increase in tumor-infiltrating NK cells in mice fed a KD. NK cells are another unique subset of the innate immune system, with several properties of the adaptive immune system [47]. Classically, they provide rapid defense against viral infection and malignant tumors. Apart from their classical anti-tumorigenic role, they also have been shown to have pro-tumorigenic and angiogenic properties [48,49]. Lussier et al. [50] demonstrated similar findings in the GL261 glioma model. Previous studies have illustrated that induction of PPARγ in NK cells induces an inhibitory effect similar to that seen in M2 macrophages [51]. However, further investigations are required to uncover the complex signaling machinery associated with PPARγ, affecting the activity of various immune cells.

## 5. Conclusions

In summary, the KD demonstrates promising anti-tumor activity in a variety of preclinical tumor models, including GBM. Utilizing the KD represents a particularly attractive strategy in GBM, as it is not influenced by the BBB, which has limited the application of an overwhelming majority of promising systemic agents in this tumor. Despite being studied as an anti-cancer strategy for over a decade, its underlying mechanism of action is still not understood and its global impact on the overall metabolism of a tumor, the TME, and the host in general is still underappreciated. In this study, we demonstrate that a KD skews macrophage polarization towards a paradoxical “pro-tumor” immune-suppressive response. Mitigating this immune response potentiated the anti-tumor activity of the KD in GBM, providing a rational combinatorial strategy that warrants further investigation.

## Data Availability

Data can be requested from the corresponding author (prakash.chinnaiyan@beaumont.edu).

## Supplementary Materials

The following supporting information can be downloaded at: https://www.mdpi.com/article/10.3390/cancers14225550/s1, Supplementary Figures S1–S4 and supplementary methods. References [22,23,24,34] are also cited in the supplementary materials.
