# Supplementary material for: The Influence of the Ketogenic Diet on the Immune Tolerant Microenvironment in Glioblastoma
Source: Cancers (Basel). 2022 Nov 11;14(22):5550. doi: 10.3390/cancers14225550 (PMC9688691; doi:10.3390/cancers14225550)
Supplement: Supplementary file 1 [file cancers-14-05550-s001.zip › cancers-1960614-supplementary.pdf]

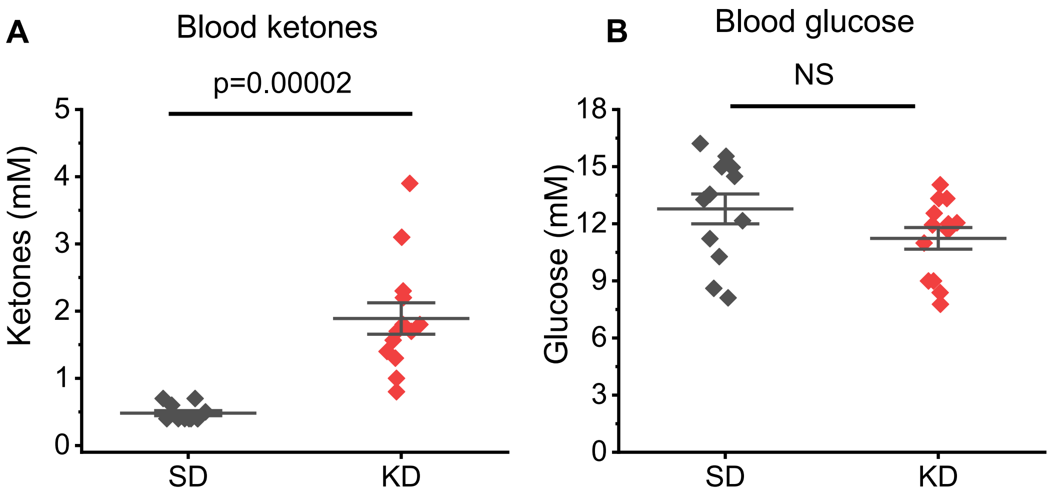


**Supplementary Figure S1:** TRP GBM tumors were grown orthotopically in immune-competent C57BL/6 mice fed a standard rodent diet (SD) or a ketogenic diet (KD) *ad libitum*. A few microliters of blood were obtained on day 10 from the start of KD (n=12). (**A**) Ketones and (**B**) glucose were measured using a hand-held glucometer. Blood glucose levels to ketone levels were plotted as a dot plot. Whiskers represent SE. N= 15/group. NS = p value >0.05.

**Supplementary Figure S1**

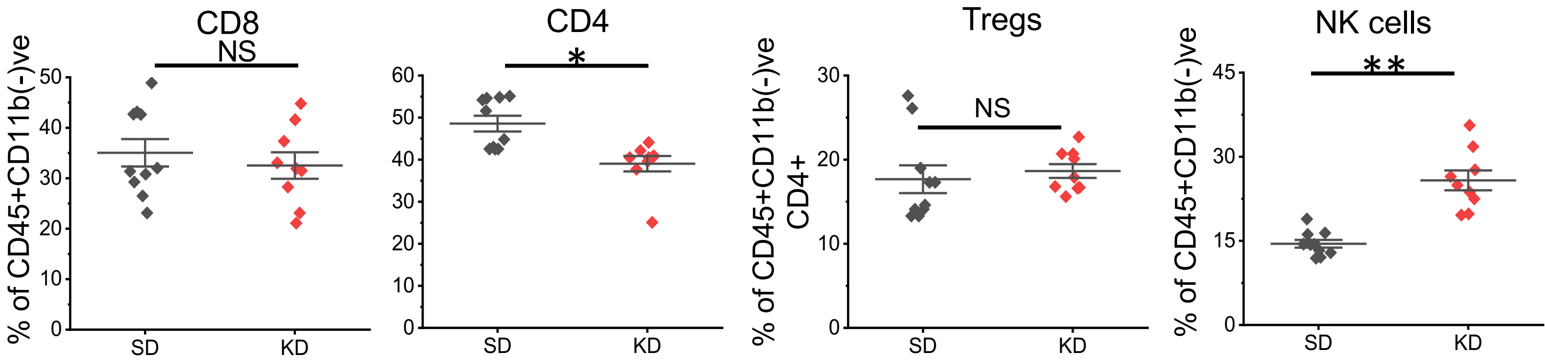


**Supplementary Figure S2:** TRP tumors were grown orthotopically in immune-competent C57BL/6 mice fed a standard rodent diet (SD) or a ketogenic diet (KD) *ad libitum*. Tumors were extracted from these mice on day 25 and analyzed for lymphocytes such as CD8+ T cells, CD4+ T cells, T regulatory (CD45+CD4+CD25+FoxP3+) cells, and NK cells (CD45+CD11b+NK1.1+). The line between the data points represents the mean, and the whisker represents SE. Scatter plot data represent N=9-10/group. NS= p >0.05, * = p<0.05 and ** = p<0.005.

# Supplementary Figure S2

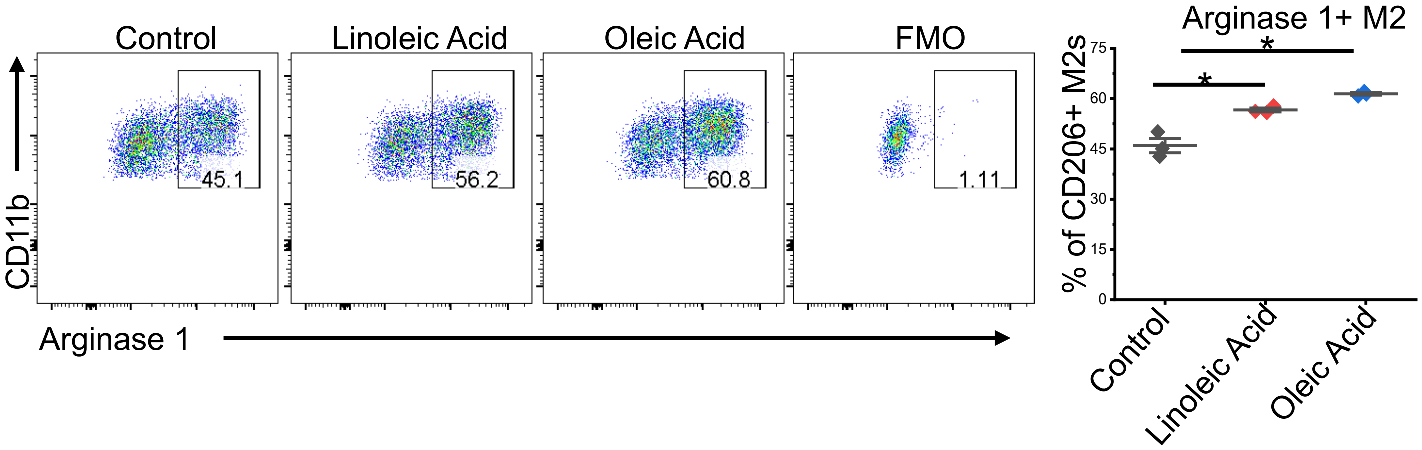


**Supplementary Figure S3:** Myeloid cells were obtained from the bone marrow of C57BL/6 mice and polarized towards M2 macrophages ± the indicated unsaturated fatty acid (linoleic acid [LA] 200 mM; oleic acid [OA] 200 μM) and analyzed by flow cytometry for Arginase 1+ M2 macrophage (CD45+CD11b+F4/80+CD206 +Arginase 1+). Numbers represents mean±SD from a minimum of 3 different experiments. The line between the data points represents the mean, and whisker represents SE. n=3/group. * = p<0.05.

# Supplementary Figure S3

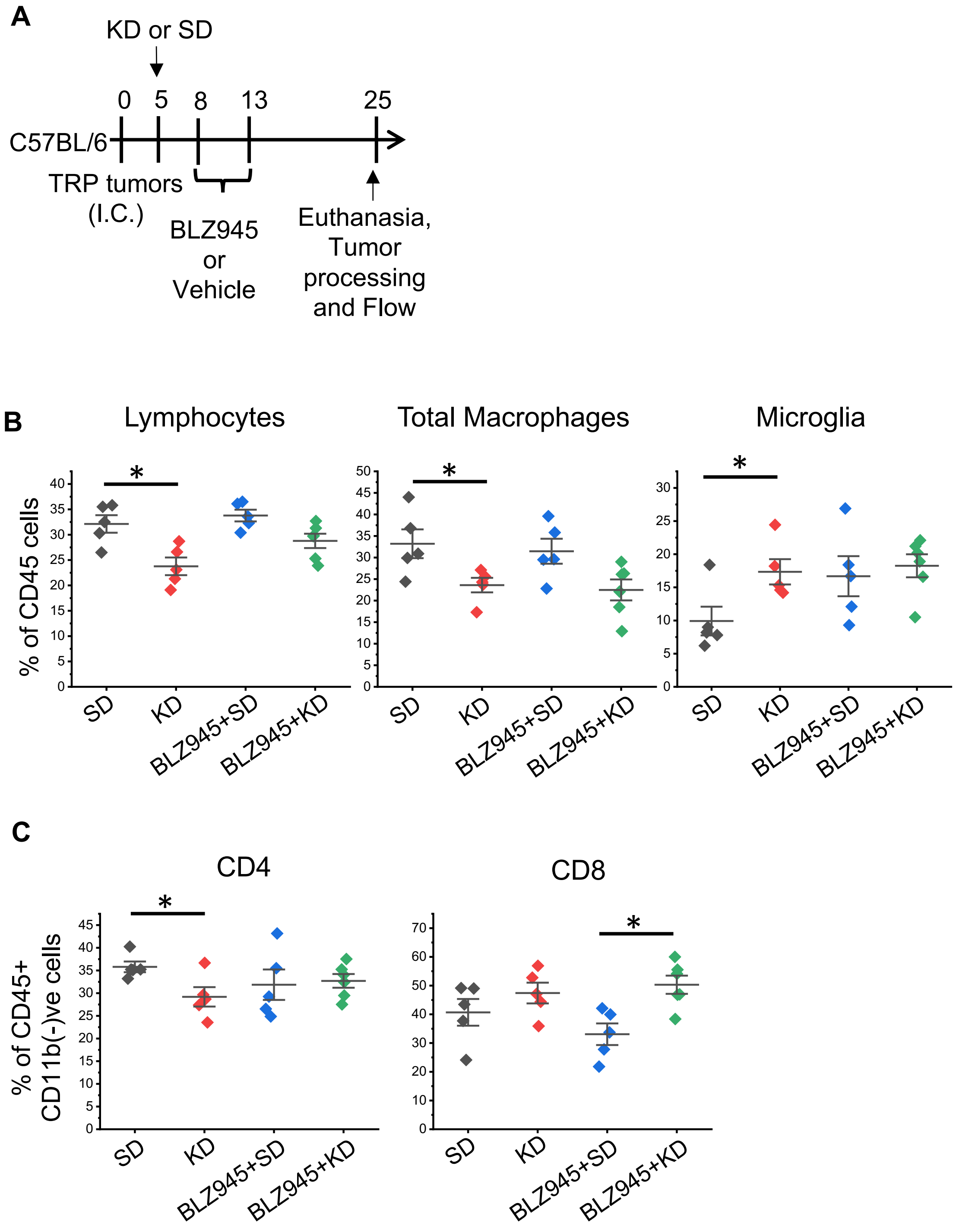


**Supplementary Figure S4:** (**A**) The schema used for correlative studies. (**B**) Tumors were harvested on day 25 from mice fed a KD or SD ± CSF-1 inhibitor BLZ945. Cells from these tumors were immunophenotyped, evaluating for total lymphocytes (CD45+CD11b-ve), total macrophages (CD45+CD11b+) and microglial cells (CD45$^{Medium}$CD11b+),(**C**) CD4+ T cells (CD45+, CD4+), and CD8+ T cells (CD45+ CD8+). The line between the data points represents the mean, and whisker represents SE. n=5-6/group. * = $p<0.05$.

**Supplementary Figure S4**

## SUPPLEMENTARY MATERIAL AND METHODS

### Reagents and antibodies

All cell culture reagents were purchased from GIBCO/Thermo (Grand Island, NY) and Corning Life Sciences (New York, NY). All cytokines for murine macrophage polarization (mIL-4, mIL-13, mIFN-$\gamma$, hIL-2, and CSF) were purchased from PeproTech (Rocky Hill, NJ). Lipopolysaccharide (LPS from E. Coli O111:B4), linoleic acid (LA), and oleic acid (OA) were purchased from Sigma Aldrich (St. Louis, MO). PPAR$\gamma$ inhibitor (GW9662) was obtained from Cayman chemicals (Ann Arbor, MI). BLZ-945 (MedKoo Biosciences, Morrisville, NC, USA) was used as a colony-stimulating factor-1 receptor (CSF-1R) inhibitor. BLZ-945 was dissolved in 20% captisol, a cyclodextrin (San Diego, CA, USA). Complete RPMI-1640 was prepared by adding heat-inactivated FBS (10%), sodium yyruvate (1mM), 2-mercaptoethanol (50$\mu$M), L-glutamine (2mM), and HEPES (25mM) buffer to RPMI-1640. Antibodies were purchased from Biolegend or eBioscience/Thermo. Clones for antibodies used for flow cytometry are as indicated: CD45 (30-F11), F4/80 (BM8), CD11b (M1/70), CD206 (C068C2), Arginase 1(A1exF5), CD80 (16-10A1), Gr1 (RB6-8C5), FoxP3 (150D/E4), CD8 (53-6.7), CD4 (Gk1.5), GzmB (QA16A02), iNOS (CXNFT), NK 1.1 (PK136) and NKG2D (C7).

### Orthotopic tumor models

We used genetically engineered mice (GEM) tumors for our orthotopic tumor model, these tumors are known as TRP. TRP tumors express a truncation mutant of SV40 large T antigen (T) from the human Gfap promoter that inactivates all 3 Rb family proteins, a constitutively active KrasG12D mutant (R), and/or a homozygous Pten deletion (P) [22-24]. Orthotopic murine TRP ($1x10^5$ cells/mouse) tumors were implanted in 6-7-week old C57BL/6 mice as previously described [22,34]. A group of mice was given KD on day 5 after the tumor implant. The remaining

mice received standard rodent chow. Mice were imaged 6-8 days after implant using Multihance (Bracco Diagnostics, Cranbury, NJ, USA) diluted in sterile saline delivered via tail vein injection under anesthesia. Mice with established tumors were used for further study. MR imaging was performed as previously described [22]. Tumor volumes were determined by delineating areas of contrast enhancement on the T1 weighted fast spin-echo sequence using the image processing PBAS tool in PMOD software (PMOD Technologies, Zurich, Switzerland). Mice were re-imaged after 2-3 weeks and followed until endpoint criteria (onset of neurologic impairment or >20% weight loss) were met. Mice were randomized into four groups of treatment vehicle alone (20% captisol), BLZ-945 alone, KD, or KD plus BLZ-945. BLZ-945 was delivered via oral gavage in 20% captisol at 200 mg/kg daily for 10 treatments [34].

**PPARγ transcriptional activation**

Nuclear proteins were extracted using a nuclear extraction kit (Cayman Chemicals, Ann Arbor, MI) from M2 macrophages or M2 macrophages polarized in the presence of LA or OA. PPARγ transcription factor ELISA assay was used to analyze PPARγ binding activity according to the manufacturer's protocol (Caymen Chemicals, Ann Arbor, MI).

**CD8 T cell suppression assay**

$CD8^+$ T cells were isolated from splenocytes by negative isolation using the mouse naïve CD8+ T cell isolation kit Dynabead (Grand Island, NY). T cells were stained with CFSE CellTrace (GIBCO/Thermo; Grand Island, NY) and activated with plate-bound α-CD3 (1μg/ml; clone- 145-2C11) and α-CD28 (5μg/ml; clone- 37.51) antibodies (eBioscinces/ Thermo; Grand Island, NY). For suppressing proliferation, polarized M2 macrophages were added at varying T cell to macrophage ratios (1:0; 2:1 and 5:1) along with 200μM of linoleic acid (LA) or oleic acid (OA). PPARγ inhibitor (GW9662) was added at the concentration of 0.5 μM

during the suppression assay. CFSE dilution on $CD8^+$ T cells was analyzed after three days of activation using a FACS Canto II flow cytometer (Becton Dickinson, Mountain View, CA).

**Flow cytometry**

Tumors were collected from mice and were lightly homogenized using DNase I and Collagenase IV (Sigma Aldrich, St. Louis, MO) to obtain a cell suspension. Cells were passed through a cell strainer to obtain a single-cell suspension. *In vitro* cultures were washed with PBS and passed through a cell strainer to obtain a single-cell suspension. Equal amount of cells/sample was used for stanning. Cell suspension was incubated with mouse Fc block (BD, Franklin Lakes, NJ). Cells were then stained for surface antigen followed by fixation and permeabilization using eBioscience transcription staining buffer (eBioscinces/ Thermo; Grand Island, NY). Further, cells were stained for intracellular/ nuclear proteins. An equal number of cells from each sample were acquired on FACS Canto II flow cytometer (Becton Dickinson; Mountain View, CA). Analysis of flow cytometry data was performed using FlowJo V10 software (FlowJo, LLC; Ashland, OR). *Gating strategy:* We use expression of CD11b and/or CD45 for initial gating of three immune subsets (i) macrophages (CD45+CD11b+), (ii) lymphocytes (CD45+CD11b-ve), and (iii) microglia ($CD45^{Medium}$CD11b+). Using initial macrophage gate we further analyzed M2 macrophages (CD45+CD11b+ F4/80+CD206+$CD80^{Low}$), Arginase 1+ M2 macrophages (CD45+CD11b+ F4/80+CD206+Arginase 1+), M1 macrophages (CD45+CD11b+ F4/80+CD206-ve,$CD80^{High}$), iNOS+M1s (CD45+F4/80+CD11b+CD206-ve,CD80hi+iNOS+), and MDSCs (CD45+ CD11b+ Gr1+). In lymphocyte gates we analyzed CD4 T lymphocytes (CD45+CD4+), CD8 T lymphocytes (CD45+CD8+), Tregs (CD45+CD4+CD25+FoxP3+), CD8+GzmB+ T lymphocytes (CD45+CD8+GzmB+), and NK cells (CD45+NK1.1+NKG2D+). For microglia analyzed $CD45^{medium}$,CD11b+CD68+ markers.

**Glucose and ketone analysis**

Blood was extracted from mice from a submandibular vein on day 10 of the start of the diet. Blood glucose and ketones were measured using Precision Xtra meter and blood glucose and β-keton test strips (Abbott Diabetes Care Inc., Alameda, CA).

**Western blot**

Western blot was performed using methods previously described.[1] PPARγ (C26H12) Cell Signaling Technology. β-Actin (ACTIN05-C4) antibodies were obtained from Thermo Fisher.
